# Applications of episodic future thinking in patients with chronic diseases: a scoping review

**DOI:** 10.3389/fpubh.2026.1914394

**Published:** 2026-09-28

**Authors:** Ranran Song, Nana Chen, Xinru Huang, Haoran Huang, Yulong Wang, Tingting Huang, Muwei Zhu, Xianping Tang

**Affiliations:** 1 School of Nursing, Xuzhou Medical University, Xuzhou, Jiangsu, China; 2 School of Management, Xuzhou Medical University, Xuzhou, Jiangsu, China

**Keywords:** chronic disease, delay discounting, episodic future thinking, intervention, scoping review

## Abstract

**Background:**

Patients with chronic diseases require long-term self-management, but adherence and sustained behavior change remain major challenges in nursing practice. Episodic future thinking (EFT) is a cognitive strategy that may influence intertemporal decision-making and health-related behaviors by increasing the salience of future outcomes. However, its application, implementation, and clinical translation across chronic disease populations have not been comprehensively synthesized. Therefore, this scoping review aimed to map the use of EFT among adults with chronic diseases, characterize its intervention components and delivery models, and examine the extent to which existing evidence extends beyond short-term cognitive and decision-making outcomes to include health behaviors and clinically relevant outcomes, thereby informing future research and clinical translation.

**Methods:**

This scoping review was conducted following PRISMA-ScR reporting guidelines. We searched PubMed, Web of Science, Embase, CINAHL, PsycINFO, The Cochrane Library, CNKI, WANFANG DATA, VIP, and SinoMed from database inception through October 2025. The methodological quality of the included studies was appraised using the 2018 Mixed Methods Appraisal Tool (MMAT).

**Results:**

Twenty studies were included, primarily involving populations with metabolic conditions; few focused on breast cancer, heart failure, or chronic pain. EFT interventions were grouped into two types: general EFT and goal-oriented EFT. Most intervention protocols included three core components: cue generation, cue appraisal, and episodic imagery training. Cue design, intervention duration, training frequency, and delivery modality varied considerably. Outcomes were categorized into four domains: decision-making and cognitive function, health-related behaviors, psychological well-being and motivation, and clinical and physiological outcomes. Evidence was strongest for proximal cognitive outcomes, particularly delay discounting, whereas behavioral outcomes were examined primarily in relation to dietary behaviors. Evidence regarding other self-management behaviors and clinically relevant outcomes was comparatively limited.

**Conclusion:**

Existing evidence suggests that EFT has potential for application in populations with metabolic conditions, but its applicability to other chronic diseases requires further validation. Overall, the field remains in transition from experimental exploration to clinical translation. Future research should focus on optimizing intervention protocols for specific diseases and populations, identifying essential intervention components, extending intervention and follow-up durations, and evaluating the feasibility and effectiveness of digital delivery approaches within chronic disease management.

**Systematic review registration:**

OSF registration: https://osf.io/rq9z7/.

## 1 Introduction

Chronic diseases are broadly defined as conditions that persist for at least 1 year and require ongoing medical attention, limit activities of daily living, or both. With increasing life expectancy, the global burden of chronic diseases has continued to rise, making them a major public health challenge worldwide (1). Approximately 41 million people die from chronic diseases every year, accounting for 74% of all global deaths (2). Beyond their impact on mortality, chronic diseases substantially reduce individuals’ functional capacity and social participation, impair quality of life, and impose considerable financial burdens on patients, families, and healthcare systems (3). The onset and progression of chronic diseases are influenced by multiple risk factors (4), among which unhealthy lifestyles—including tobacco use, poor dietary patterns, physical inactivity, and inadequate weight management—are particularly prominent (5). Consequently, the World Health Organization has identified the promotion of healthy lifestyles and the modification of harmful behaviors as among the most effective and cost-efficient strategies for chronic disease prevention and control (6). Although lifestyle interventions can encourage healthier eating and increased physical activity, their effects are often modest and difficult to sustain. Patients are often required to engage in repetitive and effortful training while continuously modifying long-established habits. This process entails an ongoing tension between immediate impulses and sustained cognitive control, which contributes to poor long-term adherence and limited intervention effectiveness (7). Accordingly, emerging evidence highlights rational health decision-making as a critical mechanism for behavior change and a promising pathway for improving health outcomes in chronic disease management (8).

Behavioral economics suggests that individuals tend to discount future rewards when making intertemporal choices, a phenomenon known as temporal or delay discounting (9). Specifically, when confronted with a trade-off between immediate pleasure and future health benefits, individuals often favor short-term gratification—such as smoking, excessive alcohol consumption, or overeating—while underestimating the long-term consequences for health and quality of life. In chronic disease management, individuals frequently face intertemporal decisions that involve trade-offs between immediate gratification and future health benefits. Given the long-term nature of chronic diseases and the delayed benefits of health-promoting behaviors, individuals may be more likely to prioritize immediate rewards. This present-oriented bias may further increase health risks and limit the real-world effectiveness of behavior-change interventions. To address this challenge, health-promotion strategies should consider individuals’ preferences for immediate versus delayed rewards to improve both initial engagement and long-term behavioral maintenance (10).

Episodic future thinking (EFT) is among the most extensively studied interventions targeting intertemporal decision-making and was first conceptualized by Atance and O’Neill (11). Also referred to as *episodic future simulation* or *episodic foresight*, EFT denotes the capacity to vividly imagine and mentally simulate personally relevant future events. Grounded in the principles of behavioral economics and positive psychology (12), EFT encourages individuals to construct detailed future scenarios, thereby rendering delayed outcomes of current health behaviors more salient, emotionally engaging, and subjectively real. Unlike traditional interventions that primarily rely on knowledge transfer or behavioral training to promote healthy behavior change, EFT influences present health decisions by modifying how individuals cognitively process future outcomes. This approach addresses the decision-making challenges associated with high delay discounting among individuals with chronic diseases and represents a promising intervention to reduce the preference for immediate gratification, promote more rational health-related decision-making, and facilitate the adoption of long-term health behaviors (Figure 1).

**Figure 1:**
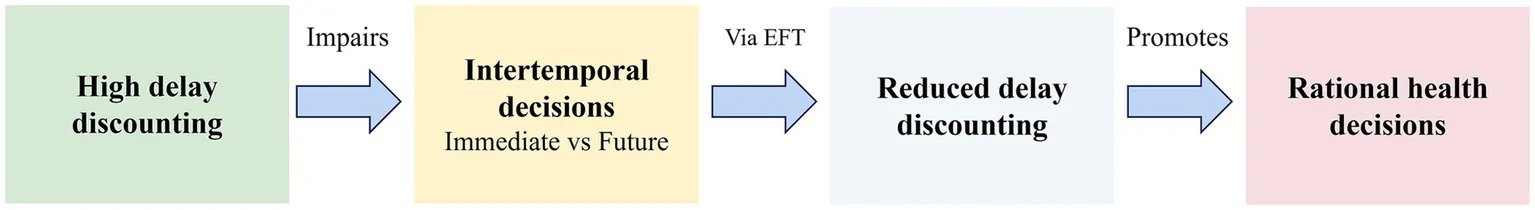
The process of EFT.

EFT is a complex mental skill that involves four components: imagining a scenario, predicting its outcome, forming an intention, and developing a plan to achieve that outcome (12). By engaging normative cognitive and neural processes, EFT facilitates healthier decision-making and behavior change. Central to EFT is the construction of personally relevant future scenarios within a specified temporal horizon. Drawing on episodic memory, this process flexibly recombines elements of autobiographical experience to generate novel, vivid, and coherent representations of possible future events (13). At the neural level, the elaboration of future scenarios engages the brain’s simulation network, particularly the hippocampus and medial prefrontal cortex. The hippocampus supports scene construction, while the prefrontal cortex evaluates the personal relevance and subjective value of anticipated outcomes, thereby enhancing the perceived importance of future rewards (12, 14). At the affective and motivational levels, EFT influences decision-making by eliciting anticipated emotions associated with future states (15). In the context of long-term health decisions, such positive future-oriented emotions can strengthen self-regulatory capacity and promote choices aligned with future well-being. Moreover, EFT has been shown to enhance psychological continuity between the present and future self (16), enabling individuals to more clearly perceive the causal link between current behaviors and downstream health outcomes. This strengthened future-self connection increases perceived responsibility and commitment to long-term health goals. Although the mechanisms through which EFT influences delay discounting and health behavior remain unclear, evidence indicates that EFT reduces delay discounting, improves decision quality, and enhances executive functioning, motivation, and mood, thereby increasing the effectiveness of health-related behavioral interventions (17).

As research on EFT has advanced, reviews have gradually expanded from examining its effects to exploring intervention methods and applications in health-related behaviors. Meta-analyses by Ye et al. and Rösch et al. showed that EFT can reduce delay discounting and promote greater consideration of long-term outcomes in decision-making (18, 19). From a methodological perspective, Brown and Stein summarized cue generation, content design, control conditions, and delivery methods for EFT interventions (20). In the health behavior field, Liu et al. reviewed EFT use in populations with overweight or obesity (21), Ding et al. evaluated its effects on weight-loss-related health behaviors and outcomes (22), and Colton et al. further quantified its effects on delay discounting and food choice (23). Overall, existing reviews suggest that EFT may reduce delay discounting, improve food choice, and reduce energy intake. However, the evidence remains concentrated in populations with overweight or obesity and in weight-management contexts, with most studies focusing on short-term cognitive and dietary outcomes. In contrast, chronic disease management requires long-term self-regulation and the maintenance of multiple health behaviors, while existing studies suggest that EFT may have broader potential across health-related behaviors (24). Building on previous reviews, this study employs a scoping review approach to systematically examine the applications and characteristics of EFT interventions across chronic disease management contexts and to evaluate the extent to which proximal cognitive effects, such as reductions in delay discounting, translate into changes in health behaviors, self-management, and clinically relevant outcomes. The review also identifies gaps in the evidence base and barriers to clinical translation, aiming to inform the design, optimization, and future implementation of EFT interventions in chronic disease management.

## 2 Methods

To map the existing evidence on the application of EFT in chronic disease management, we conducted a scoping review examining target populations, intervention components, implementation models, assessed outcomes, and key patterns and gaps in the literature. A scoping review was considered appropriate because this approach is well suited to complex and emerging areas of research, allowing for systematic characterization of the scope, distribution, and features of available evidence while identifying important knowledge gaps (25). We selected this approach rather than a systematic review because our objective was to characterize how EFT has been applied in chronic disease management and identify areas requiring further investigation, rather than estimate a pooled effect for a specific outcome or determine whether the available evidence supports or contradicts current practice (26). This study followed the scoping review framework proposed by Arksey and O’Malley (27) and comprised five stages: (1) identifying the research question; (2) identifying relevant studies; (3) study selection; (4) charting the data; and (5) collating, summarizing, and reporting results. The review protocol was registered on the Open Science Framework (https://osf.io/rq9z7/). Reporting of this review followed the Preferred Reporting Items for Systematic Reviews and Meta-Analyses extension for Scoping Reviews (PRISMA-ScR) checklist (Appendix 1).

### 2.1 Identifying the research question

Following a review of the literature and team discussions, the following research questions were formulated: (1) How are EFT interventions designed and implemented in studies involving individuals with chronic diseases or related risk states (e.g., cue development, delivery format, frequency, and duration)? (2) What outcomes have been assessed in studies of EFT interventions among individuals with chronic diseases or related risk states, and what findings have been reported? (3) What gaps exist in the current evidence base, and what recommendations can be proposed to guide future research?

### 2.2 Identifying relevant studies

To ensure comprehensive coverage of the literature, we searched multiple databases on October 15, 2025, including PubMed, Web of Science, Embase, CINAHL, PsycINFO, the Cochrane Library, and the Chinese databases CNKI, WANFANG DATA, VIP, and SinoMed. The selection of databases reflected the interdisciplinary nature of EFT in chronic disease management. PubMed, Embase, and CINAHL were used to identify studies related to medicine, nursing, and health management; PsycINFO was used to identify studies on cognitive and behavioral decision-making; and Web of Science and the Cochrane Library were used to supplement evidence from interdisciplinary and intervention research. To minimize potential language bias, we also searched Chinese databases and manually screened the reference lists of included studies to identify additional relevant literature. The search strategy combined subject headings and free-text terms, and the detailed search strategy is provided in Appendix 2.

### 2.3 Study selection

The inclusion criteria were developed based on the PCC framework (28): (1) population (P): Adults aged 18 years or older with a chronic disease or a clearly defined high-risk status for chronic disease were eligible. (2) concept (C): Studies that applied EFT as an intervention or adjunctive support strategy, defined as the use of guided imagination to simulate personally relevant future events; (3) context (C): The intervention must be implemented within the context of disease management; (4) study design: studies reporting original empirical research, including randomized controlled trials, quasi-experimental studies, or mixed-methods research; and (5) studies published in either English or Chinese.

The exclusion criteria were as follows: (1) Studies focusing on specific occupations or special populations—such as individuals with occupational diseases, disabilities, ethnic minority groups, or pregnant women—whose contextual circumstances were not aligned with the primary focus of this review; (2) Studies involving individuals with mental disorders. Because EFT relies on intact cognitive and affective processes, and mental disorders are frequently associated with alterations in executive functioning and emotional regulation, EFT interventions in these populations involve distinct mechanisms and implementation considerations that limit generalizability to other chronic disease populations (29). Therefore, interventions for individuals with mental disorders have unique characteristics and cannot be generalized to other chronic disease populations. (3) Articles classified as conference abstracts, letters, commentaries, reviews, or research protocols. (4) Gray literature, unpublished studies, or articles without full text.

Study records were managed using EndNote 21. Two researchers (RS and NC) independently screened all titles and abstracts against the predefined eligibility criteria. They then independently assessed the full texts of potentially eligible articles to determine whether the studies involved adults with chronic diseases and used EFT as an intervention or adjunctive strategy. Disagreements were resolved through discussion; when consensus could not be reached, a third researcher (XT) was consulted.

### 2.4 Charting the data

We used a standardized Excel spreadsheet to extract data on study authors, publication year, country, study design, sample characteristics, intervention methods, delivery modes, intervention frequency and duration, control conditions, outcome measures, and primary results. We also extracted EFT-related intervention characteristics, including EFT type, cue source, generation method, number of cues, emotional valence, time span, and update status (Table 1). EFT types were coded based on the definitions in Table 2 and intervention descriptions in the original studies. Cues not explicitly linked to specific goals or behaviors were classified as general EFT, whereas those targeting such goals or behaviors were classified as goal-oriented EFT (GoEFT). One researcher (RS) extracted the data, and another researcher (NC) checked them against the full texts. Disagreements were resolved through discussion; if consensus was not reached, a third researcher (XT) reviewed the original reports. Extracted data were summarized in tables.

**Table 1:** EFT approaches and cue characteristics across the included studies.

Study	EFT approach	Cue source	Cue generation method	Number of cues	Cue valence	Temporal horizon (days)	Cues updated
Brown et al. (32)	General EFT	Participant-generated	Self-guided cue generation via Qualtrics	seven	positive	30–3,650	No
Chang et al. (33)	Goal-oriented EFT	Mixed: researcher-provided goals and participant-generated personalized cues	Guided cue generation via Qualtrics	NR	positive	NR	NR
Epstein et al. (38)	Goal-oriented EFT	Participant-generated	Self-guided web-based survey	seven	positive	30–3,650	Yes—every 4 weeks
Epstein et al. (37)	Goal-oriented EFT	Participant-generated	Self-guided web-based survey	five	positive	NR	Yes—every 2 weeks
Mansouri et al. (41)	Goal-oriented EFT	Participant-generated	Self-generated and audio-recorded in the laboratory	three	positive	30–180	No; the same cues were repeated for 1 week
Stein et al. (45)	General EFT	Participant-generated	Self-guided generation task	seven	positive	1–9,125	No
Chew et al. (50)	Goal-oriented EFT	Participant-generated	Nurse-guided face-to-face activity using scripted questions	NR	positive	NR	NR
Vaughn et al. (47)	General EFT	Participant-generated	Face-to-face interview	ten	positive	7–9,125	No
Athamneh et al. (48)	General and goal-oriented EFT	Participant-generated	Self-guided online computerized task	seven	positive	30–5,840	No
Craft et al. (34)	General EFT	Participant-generated	Self-guided online generation through the MTurk study task	six	positive	7–9,125	No
Bickel et al. (31)	General EFT	Participant-generated	Self-guided generation task	three	positive	30–360	NR
Segovia et al. (43)	General EFT	Researcher-provided	digitally manipulated self-images	NR	positive and negative	NR	No
Hollis-Hansen et al. (39)	Study 1: Goal-directed process EFT	Participant-generated	Face-to-face generation through Qualtrics	three	positive	90–360	No
	Study 2: Goal-directed process EFT and general EFT	Participant-generated	Face-to-face generation through Qualtrics	three	positive	90–360	No
Hollis-Hansen et al. (40)	General EFT	Participant-generated	Face-to-face generation on a laboratory computer	one	positive	180	No
Kakoschke et al. (49)	Goal-oriented EFT	Participant-generated	Face-to-face interview	three	neutral	28	NR
Sze et al. (46)	General EFT	Participant-generated	Self-guided online generation	NR	positive	Study 1: 30–180 Study 2: 30–365	No
Stein et al. (44)	General EFT	Participant-generated	Self-guided cue generation via Qualtrics	one and three	positive	30–360	No
O'Neill et al. (42)	Goal-oriented EFT	Participant-generated	Face-to-face interview	three	positive	21	No
Daniel et al. (36)	General EFT	Participant-generated	Face-to-face laboratory task	seven	positive	1–730	No
Daniel et al. (35)	General EFT	Participant-generated	Face-to-face laboratory task	seven	positive	1–730	No

NR, not reported; positive, cues explicitly described or designed as positively valenced in the original study; neutral, cues explicitly described as emotionally neutral; Temporal horizon: interval between the present and the imagined future event; Cues updated, whether future-event cues were updated during the intervention.

**Table 2:** The types, definitions, and examples of EFT.

Types	Definition	Example
General EFT	Participants imagine non-specific, open-ended future events, such as birthdays, vacations, or family gatherings. These scenarios are positive and personalized, but do not involve specific goals.	*“In one month, I am celebrating my wife’s birthday. We are having a nice dinner with friends and family at a local restaurant. I am asking her about her year and what she is looking forward to this upcoming year. I am happy to be surrounded by friends and family.”*
Goal-oriented EFT	Individual goals are incorporated into episodic future thinking cues, where participants imagine future events related to predetermined objectives or scenarios after achieving specific health goals	*“In about 1 week, I am sitting in my room taking my medication. I am getting ready for work and sit on my bed to take my medication. I have a glass of water on the nightstand ready to use. I am alone as my husband has left for work. Next, I test my sugar level, get dressed, and leave for work. I am taking my medications to stay in good health.”*

### 2.5 Collating, summarizing, and reporting the results

In the final step, the included information was organized, synthesized, and summarized, and the results were reported as study findings. Data tables were used to categorize and summarize the data and to present key information from the included studies.

### 2.6 Quality of included articles

Although methodological quality appraisal is not required in scoping reviews, we used the 2018 Mixed Methods Appraisal Tool (MMAT) to assess the included studies and identify limitations in the existing evidence base (30). Two reviewers independently applied the MMAT criteria appropriate to each study design and rated each item as “Yes,” “No,” or “Cannot tell.” Reviewers resolved disagreements through discussion and consulted a third reviewer when they could not reach consensus. Because scoping reviews aim to map the breadth of available evidence, reviewers did not use appraisal findings to determine study eligibility, and they did not exclude any study based on methodological quality.

## 3 Results

### 3.1 Search results

A total of 985 records were initially identified through database searches. After removal of duplicates, 693 records remained for screening. Following title and abstract screening, 647 records were excluded. The remaining 46 articles underwent full-text review; 26 were excluded. Screening of reference lists did not yield any additional eligible studies. Ultimately, 20 studies met the inclusion criteria and were included in the review. The study selection process is presented in Figure 2.

**Figure 2:**
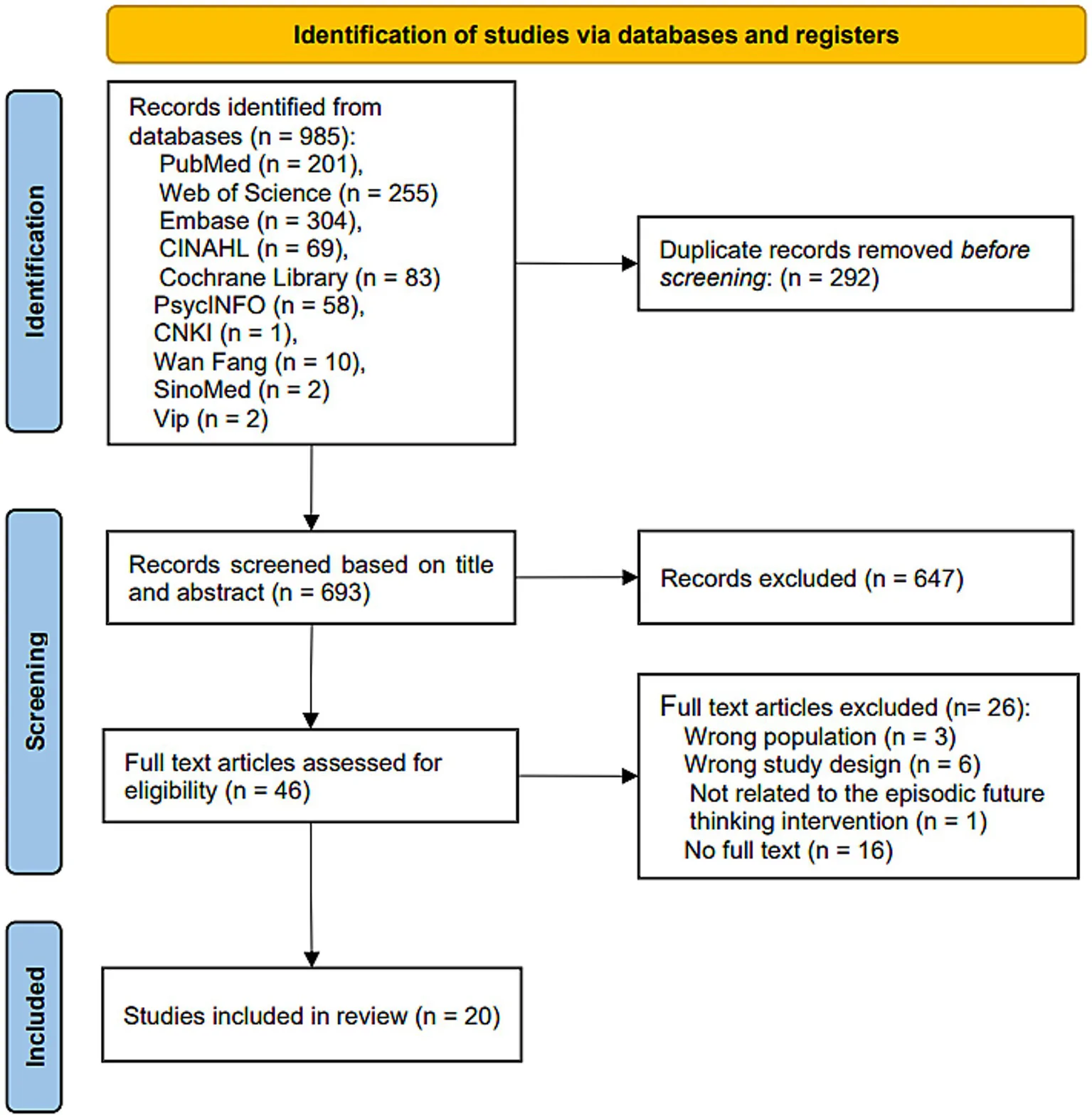
PRISMA flow diagram of study selection.

### 3.2 Characteristics of the included studies

This review included 20 studies: 18 were conducted in the United States (31–48), one in Australia (49), and one in Singapore (50). Of the included studies, 17 were randomized controlled trials; one was a single-group mixed-methods pilot pre–post study; one used a multiple-baseline design; and one used a within-subjects experimental design. Study populations comprised individuals who were overweight or obese (*n* = 13); individuals with type 2 diabetes or prediabetes accompanied by comorbid hypertension or dyslipidemia (*n* = 4); and breast cancer survivors (*n* = 1), heart failure (*n* = 1), or chronic pain (*n* = 1). Across the included studies, the sample comprised 2,264 participants, with individual sample sizes ranging from 4 to 434. Most studies included predominantly female samples, with 1,377 women (60.8%) of the total sample. Two studies did not include a control group, whereas six studies incorporated two control groups. Table 3 summarizes baseline participant characteristics.

**Table 3:** Characteristics of the included studies (*n* = 20).

Author(s) year	Country and study design	Participants	Sample characteristics	Intervention methods	Intervention provider	Delivery mode	Intervention duration and frequency	Specific control condition	Outcome measures	Main findings
Brown et al. (32)	America; RCT	Adults with type 2 diabetes and obesity	*N* = 434; mean age 44.0; M/F/Other = 162/269/3	EFT	Fully automated	Remote (Qualtrics online)	Immediate intervention	NCC HIT	DD	EFT produced significantly lower delay discounting than HIT (*p* < 0.001, d = 0.49) and NCC (*p* = 0.002, d = 0.43). No significant difference was found between HIT and NCC.
Chang et al. (33)	America; single-group mixed-methods pilot pre–post study	Low-income overweight/ obese young mothers	*N* = 15; mean age 32.9; M/*F* = 0/15	GoEFT; Online courses; Online health counseling	Research staff	Remote (Qualtrics online)	3 weeks EFT twice daily; courses/counseling weekly	None (single group)	Feasibility; Weight; Eating behavior; Emotion and stress; Motivation and self-efficacy	The program was feasible and acceptable; among completers, weight and several dietary and psychosocial indicators improved over 3 weeks.
Epstein et al. (38)	America; RCT	Prediabetes	*N* = 64; mean age 55.0; M/*F* = 13/51	EFT + BWL	Research staff	Hybrid (Face-to-face group classes and MAMRT app)	6 months 3/day in weeks 7–8, 2/day in weeks 9–14, 1/day in weeks 15–24	DCI + BWL	DD; Weight; HbA1c; Physical activity	EFT+BWL reduced cued and uncued DD more than DCI + BWL; no between-group advantage was detected for weight, HbA1c, or physical activity.
Epstein et al. (37)	America; Multiple-baseline design	diabetes with hypertension or hyperlipidemia	*N* = 4; age range 43–52; M/F/Other = 1/2/1	GoEFT	Research staff	Remote (Zoom and smartphone)	15 weeks EFT twice daily; weekly case management	None	Medication adherence; Prospective memory; DD; Working memory	Medication adherence improved overall (*p* < 0.001); prospective memory and delay discounting showed large effect sizes
Mansouri et al. (41)	America; RCT	Overweight or obese	*N* = 33; mean age 30.0; M/*F* = 14/19	GoEFT	Fully automated	Hybrid (Face-to-face interviews in the laboratory and home-based smartphone delivery)	1 week three times a day	ERT	Primary: DD; energy intake; RRV of food Secondary: NR	One week of daily EFT did not reduce delay discounting, snack-food RRV, or ad libitum energy intake compared with single EFT exposure or ERT
Stein et al. (45)	America; RCT	Overweight or obese	*N* = 78; mean age 50.4; M/*F* = 17/61	EFT	Fully automated	Face-to-face	Immediate intervention	ERT	DD; Food purchase task	EFT reduced delay discounting (*p* = 0.047), whereas scarcity increased it (*p* < 0.001), with no interaction. Neither EFT nor scarcity affected fast-food demand
Chew et al. (50)	Singapore; RCT	Heart failure	*N* = 144; mean age 61.0; M/*F* = 114/30	GoEFT Goal Setting, Action Plan, and Self-monitoring; Self-Regulation and Coping Plan	Nurse	Hybrid (face-to-face session, printed booklet and telephone follow-ups)	3 months One in-person meeting plus telephone follow-ups at weeks 3, 6, and 9	Routine care	Primary: Heart failure self-care; Secondary: consideration of future consequences, intention, behavioral automaticity, quality of life, and clinical indicators	Short-term heart-failure self-care and selected cognitive-process measures improved; secondary outcomes were nonsignificant after correction
Vaughn et al. (47)	America; RCT	Female breast cancer survivors	*N* = 89; mean age 58.69; M/*F* = 0/89	EFT	Research staff	Face-to-face	Immediate intervention	ERT	DD; Medication adherence	Higher DD was associated with poorer pill-count adherence, and EFT reduced DD versus ERT; direct post-intervention change in medication adherence was not tested.
Athamneh et al. (48)	America; RCT 2 × 2	obesity	*N* = 255; mean age 38.0; M/*F* = 104/151	EFT-general EFT-health goal	Fully automated	Remote (online computer task)	Immediate intervention	ERT-health goal; ERT-general	DD; Intensity of craving for unhealthy foods; Demand elasticity; Food craving	Both EFT conditions reduced DD. Compared with general ERT, health-goal EFT reduced fast-food demand intensity and craving and increased demand elasticity; general EFT did not significantly affect fast-food demand or craving.
Craft et al. (34)	America; RCT	Chronic pain	*N* = 250; mean age 34.9; M/*F* = 144/106	EFT	Fully automated	Remote (online computer task)	Immediate intervention	CET	DD; Severity of pain; mediation of pain change	EFT reduced DD and pain severity; the change in DD mediated the reduction in pain.
Bickel et al. (31)	America; RCT 3 × 3	Overweight or obese	*N* = 174; mean age: NR M/*F* = 89/85	EFT	Fully automated	Face-to-face computer task	Immediate intervention	Health information condition; Blank control	Food choices	Health information increased healthier snack choices; image-based EFT improved choice only among participants with obesity.
Segovia et al. (43)	America; RCT	Prediabetes with dyslipidemia and/or hypertension	*N* = 67; mean age 61.0; M/*F* = 28/39	EFT	Fully automated	Hybrid (laboratory and home web application)	NR Laboratory: one EFT session at each of the second and third visits; home practice: ≥2 times/day	CET	DD; Food shopping task; Free feeding task; Working memory	EFT reduced laboratory DD and at-home DD, but did not significantly affect food purchasing or ad libitum intake.
Hollis-Hansen et al. (39)	America; 2 × 2 RCT	Overweight or obese mothers	*N* = 60; mean age 39.2; M/*F* = 0/60	EFT + DASH EFT + Food Safety	Research staff	Remote (MAMRT app)	7–10 days EFT: At least once a day	SET: Recent mobile-gaming experience in the lab; educational control: 10-min food-safety video.	DASH diet adherence; Sodium, fat, and calorie content in household food purchases; DD	EFT improved DASH adherence; effects on sodium and fat purchases did not remain significant after correction for multiple comparisons.
Hollis-Hansen et al. (40)	America; RCT	Overweight or obese mothers	Study 1: *n* = 24; mean age 35.3; M/F = 0/24 Study 2: *n* = 33, mean age 38.3; M/F = 0/33	Study 1: EFT Study 2: general or goal/process EFT	Research staff	Face-to-face	Immediate intervention	Study 1: Savings-goal control: three non-episodic money-saving shopping cues. Study 2: ERT	Total calories from purchased food	Goal/process EFT reduced calories purchased versus savings cues (Study 1); both EFT formats reduced calories versus ERT, with no difference between EFT formats (Study 2).
Kakoschke et al. (49)	Australia; pilot RCT	Overweight or obese	*N* = 60; mean age 26.93; M/*F* = 21/39	GoEFT	Research staff	Hybrid (laboratory and smartphone)	6 weeks Once daily for 1 week	Active comparator: AAT; control: wait-list/no training	Primary: hypothetical healthy food choice; secondary: weight change; other outcomes: approach bias, DD	EFT did not significantly affect delay discounting, healthy food choice, or weight. In contrast, AAT reduced approach bias for unhealthy food, increased healthy food choice, and produced a small within-group weight reduction.
Sze et al. (46)	America; Study 1: RCT study2: 2 × 3 RCT	Overweight or obese	Study 1: *n* = 66; mean age 37.29 M/F = 21/45; Study 2: *n* = 204, mean age 37.93 M/*F* = 98/106	Online EFT	Fully automated	Remote	Immediate intervention	ERT; no episodic thinking; neutral-income narrative	Study 1: DD Study 2: DD; Food demand	Online EFT reduced DD in both experiments and reduced fast-food demand even under negative income shock.
Stein et al. (44)	America; RCT	Overweight or obese	*N* = 131; mean age 35.6; M/*F* = 57/74	EFT	Fully automated	Remote (Qualtrics online)	Immediate intervention	One-cue or three-cue ERT	DD	Three-cue EFT reduced DD; a single EFT cue did not.
O'Neill et al. (42)	America; RCT	women with overweight or obesity	*N* = 29; mean age 36.7; M/*F* = 0/29	GoEFT	Research staff	Face-to-face (laboratory cue creation and smartphone cue playback in a food court)	1 day	ERT	Total energy intake; macronutrient distribution	EFT reduced total energy and the percentage of energy from fat and increased the percentage of energy from protein versus ERT.
Daniel et al. (36)	America; Within-subject experimental design	women with overweight or obesity	*N* = 24; mean age 26.2; M/F = 0/24	EFT	Research staff	Face-to-face	3–7 days	CET: Read the first two chapters of *Pinocchio* and imagined vivid story events.	DD; Time perspective; BIS/BAS	EFT reduced DD in the included overweight/obese subgroup
Daniel et al. (35)	America; RCT	women with overweight or obesity	*N* = 26; mean age 26.4; M/F = 0/26	EFT	Research staff	Face-to-face	Immediate intervention	CET	DD; Calories; Degree of imagery	EFT reduced DD and ad libitum energy intake versus ERT.

RCT, randomized controlled trial; EFT, episodic future thinking; GoEFT, goal–oriented EFT; ERT, episodic recent thinking; NCC, no-cue control condition; HIT, Health Information Thinking; DD, delay discounting; BWL, behavioral weight loss; DCI, daily check in; CET, control episodic thinking; DASH, dietary approaches to stop hypertension; SET, standardized episodic thinking; AAT, approach–avoidance training; BIS/BAS, Behavioral Inhibition System/Behavioral Activation System scales; RRV, relative reinforcing value; HbA1c, glycated hemoglobin; MAMRT, Mobile Audio Manager and Response Tracker; NR, not reported.

### 3.3 Quality of the included studies

Of the 20 included studies, 5 were rated high quality (80–100%), 11 moderate quality (60–79%), and 4 low quality (below 60%). Overall, methodological quality was moderate. Appendix 3 presents detailed appraisal results. Among the randomized controlled trials, only six reported their randomization procedures in sufficient detail (31, 38, 45, 47, 49, 50); the remainder reported random assignment but provided insufficient information on sequence generation or allocation concealment. Baseline characteristics were generally comparable, and interventions were largely delivered as intended. However, three studies reported baseline imbalances in age, income, or education (34, 43, 50), and two had incomplete outcome data (32, 49). Only six studies explicitly reported blinding of outcome assessors (38, 43–46, 50), suggesting potential outcome assessment bias. In the two non-randomized studies (36, 37), small samples, the absence of concurrent control groups, and multiple intervention components limited control of confounding and causal inference. The single-group mixed-methods pilot study (33) effectively integrated quantitative and qualitative findings, but its small sample size, lack of a control group, and limited control of confounding reduced generalizability and weakened causal inference.

### 3.4 Types and delivery process of EFT interventions

Currently, the application of EFT in individuals with chronic diseases primarily falls into two approaches: general EFT and goal-oriented EFT (see Table 2). Of the 20 included studies, 11 used only general EFT (31, 32, 34–36, 40, 43–47), seven used goal-oriented EFT (33, 37, 38, 41, 42, 49, 50), and two studies compared general EFT and goal-oriented EFT (39, 48). Across studies, the development of episodic future thinking (EFT) interventions typically involved three sequential phases. (a) Cue generation: Participants generated EFT cues through face-to-face interviews or self-guided computerized tasks, describing positive, concrete, and vivid events occurring at multiple future time points. Participants were instructed to imagine each event as if it were unfolding in the present, with an emphasis on specific sensory details and a clearly defined contextual setting. The number of cues, temporal horizons, and emotional valence varied across studies; detailed characteristics are summarized in Table 1. (b) Cue evaluation: Participants rated each cue on dimensions such as vividness, importance, likelihood, and emotional intensity (e.g., excitement). Based on these ratings, higher-rated cues were selected and retained as text- or audio-based prompts for subsequent training. (c) Training delivery: Selected cues were delivered using guided reading aloud or audio playback. In several studies, cues were periodically updated to preserve personal relevance over time.

### 3.5 Intervention characteristics of EFT

#### 3.5.1 Study population and intervention setting

Thirteen studies applied EFT in overweight or obese populations. One study employed digitally manipulated self-images to enable participants to visualize their potential future body shape, thereby priming future-oriented cognition and enhancing the salience of long-term weight-related outcomes (43). Another study focused on patients with heart failure, applying EFT to enhance self-management motivation and improve self-care behaviors (50). Four trials tested EFT in adults with type 2 diabetes or prediabetes and comorbid hypertension or hyperlipidemia (31, 32, 37, 38). One trial targeted breast cancer survivors, examining the effects of EFT on medication adherence (47), while another investigated whether EFT could modulate pain perception and health-related behaviors among individuals with chronic pain (34). Regarding setting, eight studies were conducted exclusively in laboratory environments (35, 36, 39, 43, 45–48). Nine were implemented in real-world contexts, including seven in participants’ homes (31, 33, 37, 38, 40, 41, 49), one in a hospital (50), and one in a food court (42). The remaining three imposed no location constraints (32, 34, 44).

#### 3.5.2 Intervention duration and frequency

EFT intervention duration and delivery varied substantially across studies. Ten studies delivered EFT as a single-session or momentary intervention, typically as a one-off task in a laboratory or online setting (32, 34, 35, 39, 43–48). One study did not report the overall duration of the intervention (31). The remaining nine studies reported intervention durations ranging from 1 day to 6 months. Among these studies, six explicitly required participants to practice EFT one to three times per day (33, 37, 38, 40, 41, 49), whereas the others did not specify the frequency of daily practice (36, 42, 50). Only two studies reported systematic updating of EFT cues over time. One study updated short-term future-scenario cues every 4 weeks (38), while the other updated medication-related cues every 2 weeks (37).

#### 3.5.3 Intervention delivery format

EFT interventions were delivered through three primary formats: face-to-face laboratory-based sessions, online platforms, and hybrid approaches. In seven studies, EFT was delivered in laboratory settings as a short-term, face-to-face intervention (35, 36, 39, 42, 43, 45, 47). Eight studies delivered EFT via online platforms, most commonly asking participants to generate episodic future cues through self-guided computer tasks or tools such as Qualtrics, smartphone applications, and digitally manipulated self-images (32–34, 37, 40, 44, 46, 48). The remaining five studies adopted hybrid formats in which individualized EFT cues were typically generated via face-to-face interviews, group sessions, or other offline procedures and then practiced in naturalistic settings, supported by smartphone apps, online platforms, or telephone follow-up (31, 38, 41, 49, 50).

### 3.6 Reported outcomes of EFT interventions

#### 3.6.1 Decision-making and cognitive outcomes

Across the 12 included studies, EFT was associated with improvements in intertemporal decision-making and cognitive function (31, 32, 34–38, 44–48). Several studies reported that participants receiving EFT exhibited greater self-regulatory control and a stronger future-oriented perspective when weighing immediate rewards against longer-term benefits. However, these effects varied across intervention conditions. For example, Stein et al. found that three-cue EFT was associated with reduced delay discounting, whereas a single cue had no significant effect. Similarly, Epstein et al. observed a substantial pre–post reduction in delay discounting, although the sample comprised only four participants. In contrast, other studies reported limited or nonsignificant effects of EFT. Mansouri et al. found no significant effects of either a single EFT session or 1 week of repeated EFT on delay discounting. Similarly, Kakoschke et al. found no significant effect of mobile EFT training on delay discounting for either monetary or food rewards. Beyond delay discounting, several studies assessed other cognitive and future-oriented outcomes, including working memory, prospective memory, future-oriented cognition, and consideration of future consequences. For example, Chew et al. reported that EFT enhanced future-oriented cognition and broadened temporal awareness.

#### 3.6.2 Health-related behavioral outcomes

Regarding health-related behavioral outcomes, 10 studies reported favorable effects of EFT, primarily on healthy eating behaviors. Three studies indicated that, in addition to improving decision-making and cognitive function (35, 46, 48), EFT was associated with favorable dietary outcomes, with effects also observed under experimentally simulated economic scarcity (46). However, improvements in cognitive outcomes did not consistently translate into changes in dietary behavior. Hollis-Hansen et al. reported improved DASH diet adherence following EFT but observed no significant change in delay discounting. Conversely, Bickel et al. observed reduced delay discounting but no significant changes in food purchases or free-choice intake. Four additional studies reported favorable effects of EFT on dietary behaviors (33, 39, 42, 43). Regarding other chronic disease self-management behaviors, a multiple-baseline study reported improved medication adherence following EFT (37). In breast cancer survivors, higher delay discounting was associated with poorer medication adherence, and EFT reduced delay discounting; however, post-intervention changes in medication adherence were not directly assessed (47). A study in patients with heart failure also reported short-term improvements in self-care following an EFT-based intervention (50). Only one study examined physical activity outcomes. Although EFT was associated with reduced delay discounting among adults with prediabetes, it did not lead to additional improvements in physical activity (38).

#### 3.6.3 Psychological and motivational outcomes

Two studies reported favorable outcomes related to psychological well-being and behavioral motivation following interventions incorporating EFT. In Singapore, a 12-week nurse-led self-regulation program for patients with heart failure embedded EFT within a temporal self-regulation framework. Participants constructed personalized future health scenarios and practiced emotion regulation and attention switching techniques, which were associated with marked gains in future orientation and health-behavior motivation (50). In a separate pilot trial including low-income adults with overweight or obesity in the US, researchers developed a 3-week goal-oriented EFT intervention that combined online modules with daily text prompts. Participants repeatedly revisited weight-loss-related future scenes and wrote EFT entries; the intervention was acceptable and was associated with increased autonomous motivation, greater self-efficacy, and more positive health intentions. This was also the only study to examine participants’ intervention experiences through qualitative interviews (33).

#### 3.6.4 Clinical and physiological outcomes

Clinical and physiological outcomes were rarely assessed, and the findings were inconsistent. Among individuals with chronic pain, Craft et al. reported that EFT reduced both delay discounting and pain severity, with reductions in delay discounting mediating the improvement in pain outcomes (34). A single-group pilot study of low-income young mothers with overweight or obesity also reported short-term weight reduction following a multicomponent GoEFT intervention (33). In contrast, two studies found no significant additional improvements in glycated hemoglobin (HbA1c) or body weight with EFT compared with the respective control conditions (38, 49). Overall, clinical and physiological outcomes have been less frequently examined than proximal cognitive outcomes, and the findings remain inconsistent.

### 3.7 Summary of evidence gaps

The existing evidence base has several important limitations. First, studies were concentrated primarily in populations with metabolic conditions, with comparatively limited evidence from other chronic disease groups. Women were overrepresented, older adults were underrepresented, and no studies examined sex as a potential moderator of EFT effects. Second, substantial heterogeneity was observed across studies in cue generation, cue assessment, imagery training procedures, control conditions, delivery approaches, and intervention dose. In addition, some interventions incorporated other behavioral strategies, making it difficult to isolate the specific contribution of EFT. Third, most studies evaluated single-session or short-term interventions, and long-term follow-up was uncommon, leaving the durability and maintenance of EFT effects uncertain. Fourth, outcomes were largely concentrated on proximal cognitive measures, particularly delay discounting, and dietary behaviors, whereas medication adherence, physical activity, and other chronic disease self-management behaviors were assessed less frequently. Evidence for distal clinical and physiological outcomes was also sparse. Fifth, intervention acceptability was examined in only one small mixed-methods study, and patients’ perspectives regarding intervention burden, preferences, usability, and sustained EFT engagement remain insufficiently explored. Finally, several methodological concerns were evident, including small sample sizes, limited reporting of randomization and blinding procedures, baseline imbalances, and incomplete outcome data.

## 4 Discussion

This scoping review included 20 studies published between 2013 and 2023 that examined the use of EFT among individuals with chronic diseases or those at high risk of developing chronic diseases, with a focus on intervention characteristics and associated outcomes. The included studies showed substantial heterogeneity in study design, intervention delivery, and outcome measures, suggesting that the field is still transitioning from experimental exploration to clinical validation. EFT interventions primarily fall into two types: general EFT and goal-oriented EFT. Across studies, researchers typically described the EFT cue-creation process in three phases: generation, evaluation, and training. We grouped findings from 20 studies into four outcome domains: decision-making and cognitive function, health-related behaviors, psychological well-being and motivation, and clinical and physiological outcomes. Of the 20 included studies, 12 overlapped with those included in the scoping review by Liu et al., whereas 8 were not included in that review. Among the eight studies, six examined populations with diabetes or prediabetes, heart failure, breast cancer, or chronic pain. Compared with previous reviews, which primarily focused on populations with overweight or obesity and outcomes such as delay discounting and food choice (23), the present review encompasses a broader range of chronic conditions and outcomes. It also examines, from a clinical translation perspective, how the available evidence extends beyond proximal cognitive outcomes to health behaviors, self-management, and clinically relevant outcomes.

### 4.1 Characteristics of study populations

With respect to participant characteristics, existing EFT research has focused predominantly on individuals with overweight or obesity, consistent with previous reviews and suggesting that weight management remains the primary area of application. Given the challenges these populations may face in regulating dietary intake, resisting immediate rewards, and sustaining long-term lifestyle changes, EFT-based simulation of future events may offer a useful strategy for promoting more future-oriented health decisions and supporting behavior maintenance. However, despite the expanding use of EFT in chronic disease management, evidence from other patient populations remains comparatively limited. Women were also overrepresented across the included studies. Although this imbalance was partly attributable to studies that specifically recruited mothers or breast cancer survivors, it may also reflect differential recruitment and participation patterns. Previous research has shown that men are less likely to participate in lifestyle interventions, potentially because of lower help-seeking tendencies, traditional masculine norms, perceptions that weight-management services are oriented toward women, and limited alignment between intervention formats and men’s preferences and needs (51, 52). Although some included studies considered sex in baseline comparisons or statistical analyses, none specifically examined whether sex moderated the effects of EFT. Therefore, sex differences in EFT effects and its applicability to men with chronic diseases remain unclear. Older adults were similarly underrepresented in the existing literature. This underrepresentation may partly relate to age-associated declines in episodic future thinking and prospective memory (53, 54). Notably, studies suggest that prompting older adults to generate detailed, context-rich future events may improve prospective memory and planned behavior execution (55), indicating EFT’s potential in older adults with chronic diseases.

### 4.2 Design and delivery of EFT interventions

Researchers mostly used general EFT in a single-session or short-term experiment to examine proximal cognitive outcomes such as delay discounting, whereas GoEFT was more commonly applied in real-world contexts such as medication adherence, weight management, and eating behaviors. Athamneh et al. found that while health-goal EFT did not reduce delay discounting more than general EFT, it had a greater impact on the intensity of fast-food cravings (48), suggesting its advantage may lie primarily in goal-consistent behavioral outcomes. Differences in intervention effects may also be related to cue source, number of cues, emotional valence, and time span. Most studies used participant-generated, individualized cues to enhance the self-relevance of future events. Olsen et al. found that imagining self-relevant future events was more effective in reducing delay discounting than imagining events occurring to others (56), further supporting the importance of self-relevance. Regarding cue number and updating, existing evidence suggests that multi-cue EFT may be more effective than single-cue EFT, possibly because repeated exposure to a single cue may lead to habituation; however, the effects of cue number and time span remain difficult to distinguish (44). Although two long-term studies periodically updated the cues and reported improvements in relevant outcomes, neither examined the effect of cue updating in isolation. In contrast, daily EFT repetition for 1 week did not yield additional benefits (41), suggesting that maintaining cue novelty may matter more than simply increasing repetition frequency. Most of the included studies used positive cues. Previous meta-analyses and experimental studies suggest that positive valence may enhance, rather than determine, EFT effectiveness, while neutral or negative cues may also be effective in specific contexts (19, 57, 58). Furthermore, the time spans examined across studies ranged from the near future to several years, and evidence regarding their moderating role remains inconsistent (20). Overall, current evidence supports the use of self-relevant, concrete, vivid, and target behavior–aligned cues; however, the independent contributions of each element and their optimal combinations require further validation.

Most included studies used single-session or short-term interventions in laboratory or acute experimental settings, and most evaluated EFT as a standalone intervention. The available evidence primarily supports the immediate effects of EFT, whereas whether repeated practice can build stable self-regulation skills and translate into lasting behavioral and clinical benefits remains unclear. This review further found that some studies combined EFT with behavioral weight loss, case management, or nursing support, suggesting that EFT may function as a cognitive support strategy alongside other behavioral interventions for long-term self-management of chronic conditions. Future research should explore integrating EFT into chronic disease management programs through personalized future-scenario prompts, cue updating, and ongoing training to determine its potential to sustain health behaviors and improve clinical outcomes. Some studies have also integrated EFT into an ecological momentary intervention (EMI) framework, using mobile devices to deliver personalized cues describing future events in real time during everyday activities. This approach extends EFT beyond the laboratory and may enhance its ecological validity and sustainability. Digital EFT appears feasible and may facilitate broader delivery and improved accessibility (59). However, limited digital health literacy, restricted access to devices or reliable internet, and difficulties using digital technologies may hinder participation among older adults and individuals with limited socioeconomic resources (60). Digital interventions also commonly experience declining engagement and substantial attrition. For EFT, the need to generate cues independently and practice repeatedly may increase user burden and fatigue, potentially undermining sustained, consistent training (61). Therefore, digital interventions should balance scalability with personalized support and user burden.

### 4.3 Outcomes associated with EFT interventions

Overall, EFT shows potential for improving decision-making and cognitive function, health behavior adherence, psychological well-being and motivation, as well as selected clinical and physiological outcomes; however, findings remain heterogeneous across studies. EFT engages the hippocampus and medial temporal lobe systems to construct specific future scenarios, while the medial prefrontal cortex integrates their anticipated emotional and subjective value into current decision-making, thereby extending the decision-making time window. Its effect on delay discounting may therefore be particularly direct. Existing research suggests that EFT’s effects on delay discounting may be influenced by training duration, cue updating, and whether cues are presented during assessment. A one-week ecological momentary intervention improved DASH diet adherence but did not alter delay discounting under unprompted conditions, suggesting that short-term training may be insufficient to establish future-oriented thinking as a stable default decision-making pattern (40). Similarly, repeated use of the same EFT cues for 1 week did not yield additional improvements, possibly because of habituation from repeated exposure (41). In a smartphone-based intervention, the interval between training and subsequent cue-free assessment, together with limited cue vividness, may also have weakened the effect (49). In contrast, a 24-week structured intervention reduced delay discounting even under cue-free conditions, suggesting that sustained training may help generalize EFT effects to decision-making without external cues (38). Thus, cue-present assessments may primarily reflect the immediate priming effects of EFT cues, whereas cue-free assessments may depend more on sustained training and intervention adherence.

When cues appear at key decision points and align with the target behavior, EFT effects may extend to behavioral outcomes such as food choice. However, some studies reported reductions in delay discounting without corresponding improvements in food purchases, energy intake, or fast-food cravings. Similarly, Colton et al.’s meta-analysis showed larger effects of EFT on delay discounting than on food choice, suggesting that intervention effects may diminish as outcomes become more distal to the core cognitive mechanism (23). More complex self-management behaviors, such as medication adherence, involve multiple factors, including prospective memory, behavioral habits, and context. Goal-oriented EFT may facilitate these behaviors, but current evidence is limited to small-sample studies (37). Studies in patients with heart failure suggest that comprehensive self-regulation interventions incorporating EFT activities may improve self-care behaviors; however, integrating multiple behavior change strategies makes it difficult to isolate EFT’s specific contribution (50). Notably, the 24-week trial did not demonstrate additional improvements in weight or HbA1c, indicating that proximal cognitive changes alone may be insufficient to produce clinical benefits. Clinical and physiological outcomes represent distal outcomes in the EFT pathway. Previous behavioral intervention research suggests that physiological improvements may emerge gradually and also depend on baseline metabolic status, intervention intensity, and ongoing support (62). Therefore, whether EFT can produce clinically meaningful physiological benefits remains uncertain, and evidence for these outcomes is the most limited among outcome domains.

### 4.4 Future research directions

Future research should include a broader range of chronic conditions and improve sample representativeness through targeted recruitment and predefined age and sex stratification. This would facilitate a better understanding of population heterogeneity and the long-term effects of EFT. Given the substantial heterogeneity in cue characteristics, intervention dose, and delivery methods across existing studies, direct comparisons, factorial designs, or other component analysis approaches are needed to identify optimal intervention strategies. Future studies should also explore goal-oriented EFT to better address long-term self-management needs and optimize cue characteristics and updating procedures to improve intervention sustainability and applicability. The practical value of EFT requires further validation through studies with adequate sample sizes, rigorous designs, and longer follow-up periods. These studies should examine whether sustained behavioral changes translate into improvements in clinical and physiological outcomes. Appropriate control conditions should be incorporated to clarify the independent effects of EFT and evaluate its added value when integrated with broader chronic disease management strategies, such as action plans, contextual reminders, and ongoing training. In addition, qualitative or mixed-methods approaches should be used to examine perceived burden, preferences, and user experiences during sustained use and to optimize intervention design based on participant feedback. For older adults and individuals with limited digital literacy, age-friendly and low-burden designs should be prioritized. Digital skills training, process monitoring, and professional support should be provided when needed. Flexible cue formats, including images, text, or synthetic speech, should be tailored to individual preferences and usage contexts to improve the feasibility of digital EFT interventions in chronic disease management.

## 5 Limitations

This review has several limitations. First, the existing evidence is concentrated largely among individuals with overweight or obesity, who were represented in 13 of the 20 included studies, resulting in substantial overlap with populations examined in previous reviews. Accordingly, the present review primarily maps the scope and distribution of EFT evidence across chronic disease management contexts rather than establishing its effectiveness across different chronic disease populations. In addition, individuals with mental disorders were not included. As specified in our eligibility criteria, mental disorders were excluded from the definition of chronic diseases used in this review because EFT interventions for these conditions may involve distinct cognitive and emotional mechanisms and implementation characteristics, limiting comparability with other chronic disease populations. Second, most studies used single-session or short-term interventions with limited follow-up. Current evidence primarily reflects short-term cognitive and behavioral outcomes, whereas the long-term effects of EFT on self-management and clinical outcomes remain uncertain. In addition, studies from low- and middle-income countries are lacking, and EFT effects and implementation may vary across cultural contexts. Cross-cultural research is therefore needed to examine its applicability across diverse populations. Finally, although this study employed a comprehensive search strategy, potential selection bias cannot be completely ruled out due to language restrictions, database selection, and predefined search terms; some relevant studies may have been missed.

## 6 Conclusion

This scoping review summarizes study populations, intervention components, delivery approaches, and outcomes reported in studies of EFT for chronic disease management, providing an overview of the current evidence base and its potential applications. Current evidence suggests that EFT may influence decision-making and cognitive function, health-related behaviors, psychological well-being and motivation, and selected physiological outcomes; however, the strength and consistency of evidence vary considerably across outcome domains. Evidence is strongest for proximal cognitive outcomes, particularly delay discounting, more limited for health-related behaviors, and sparse for clinically relevant and physiological outcomes. Overall, EFT shows potential as a flexible, individualized, and digitally adaptable cognitive self-regulation strategy for chronic disease management; however, its optimal intervention model and long-term health effects require further investigation. Future research should prioritize adequately powered randomized controlled trials with rigorous designs, more diverse populations, and longer intervention and follow-up periods. Studies should also identify core intervention components and optimal delivery protocols and determine whether improvements in proximal cognitive outcomes translate into sustained changes in self-management behaviors and clinically meaningful benefits. In addition, qualitative or mixed-methods approaches should be used to assess intervention acceptability and explore participant experiences, while the role of digital EFT interventions in chronic disease management warrants further investigation.

## Data Availability

The original contributions presented in the study are included in the article/Supplementary material, further inquiries can be directed to the corresponding authors.
