# Supplementary material for: Applications of episodic future thinking in patients with chronic diseases: a scoping review
Source: Front Public Health. 2026 Sep 28;14:1914394. doi: 10.3389/fpubh.2026.1914394 (PMC13655094; doi:10.3389/fpubh.2026.1914394)
Supplement: Supplementary file 1 [file Data_Sheet_1.docx]

# **Appendix 1 Search syntax**

## **Database 1: PubMed**

(("Chronic Disease"[Mesh] OR "Noncommunicable Diseases"[Mesh] OR "chronic illness"[Title/Abstract] OR "chronic condition"[Title/Abstract] OR "noninfectious disease*"[Title/Abstract] OR "Cardiovascular Diseases"[Mesh] OR "Hypertension"[Mesh] OR "High Blood Pressure"[Title/Abstract] OR "Coronary Disease"[Mesh] OR "Coronary Artery Disease"[Mesh] OR "heart valve disease*"[Title/Abstract] OR "heart disease*"[Title/Abstract] OR "Stroke"[Mesh] OR "Cerebrovascular Accident"[Title/Abstract] OR "Pulmonary Disease, Chronic Obstructive"[Mesh] OR "Asthma-Chronic Obstructive Pulmonary Disease Overlap Syndrome"[Mesh] OR "Asthma"[Mesh] OR "COPD"[Title/Abstract] OR "Chronic obstructive pulmonary disease"[Title/Abstract] OR "Chronic Obstructive Lung Disease"[Title/Abstract] OR "Chronic Bronchitis"[Title/Abstract] OR "Pulmonary Arterial Hypertension"[Title/Abstract] OR "Pulmonary Heart Disease"[Title/Abstract] OR "Metabolic Syndrome"[Mesh] OR "Diabetes Mellitus"[Mesh] OR "Diabetes Mellitus, Type 1"[Mesh] OR "Diabetes Mellitus, Type 2"[Mesh] OR "Obesity"[Mesh] OR "Diabetes"[Title/Abstract] OR "Neoplasms"[Mesh] OR "tumor*"[Title/Abstract] OR "cancer*"[Title/Abstract] OR "malignant tumor*"[Title/Abstract])) AND (((((((("episodic future thinking"[Title/Abstract]) OR ("episodic simulation"[Title/Abstract])) OR ("episodic prospection"[Title/Abstract])) OR ("mental time travel"[Title/Abstract])) OR ("prospective thinking"[Title/Abstract])) OR ("counterfactual thinking"[Title/Abstract])) OR ("foresight"[Title/Abstract])) OR ("imagining the future"[Title/Abstract]))

**Results: 201**

## **Database 2: Web of Science**

(TS=("Chronic Disease" OR "Noncommunicable Diseases" OR "Chronic Illness" OR "Chronic Condition" OR "noninfectious disease*" OR "Cardiovascular Diseases" OR "Hypertension" OR "High Blood Pressure" OR "Coronary Disease" OR "Coronary Artery Disease" OR "Heart Valve Disease*" OR "heart disease*" OR "Stroke" OR "Cerebrovascular Accident" OR "Asthma-Chronic Obstructive Pulmonary Disease Overlap Syndrome" OR "Asthma" OR "COPD" OR "Chronic obstructive pulmonary disease" OR "Chronic Obstructive Lung Disease" OR "Chronic Bronchitis" OR "Pulmonary Arterial Hypertension" OR "Pulmonary Heart Disease" OR "Metabolic Syndrome" OR "Diabetes Mellitus" OR "Diabetes type 1" OR "Diabetes type 2" OR "Obesity" OR "Diabetes" OR "Neoplasms" OR "tumor*" OR "cancer*" OR "malignant tumor*")) AND (TS=("episodic future thinking" OR "episodic simulation" OR "episodic prospection" OR "mental time travel" OR “prospective thinking” OR "counterfactual thinking" OR "foresight" OR "imagining the future"))

**Results: 255**

## **Database 3: Embase**

#1. ('chronic disease'/exp OR 'chronic disease') AND [embase]/lim

#2 ('non communicable disease':ti,ab,kw OR 'cardiovascular disease':ti,ab,kw OR 'hypertension':ti,ab,kw OR 'coronary artery disease':ti,ab,kw OR 'heart disease':ti,ab,kw OR 'cerebrovascular accident':ti,ab,kw OR 'asthma-chronic obstructive pulmonary disease overlap syndrome':ti,ab,kw OR 'asthma':ti,ab,kw OR 'chronic obstructive lung disease':ti,ab,kw OR 'chronic bronchitis':ti,ab,kw OR 'metabolic disorder':ti,ab,kw OR 'diabetes mellitus':ti,ab,kw OR 'insulin dependent diabetes mellitus':ti,ab,kw OR 'non insulin dependent diabetes mellitus':ti,ab,kw OR 'obesity':ti,ab,kw OR 'neoplasm':ti,ab,kw OR 'chronic condition':ti,ab,kw OR 'noninfectious disease':ti,ab,kw OR 'high blood pressure':ti,ab,kw OR 'coronary disease':ti,ab,kw OR 'heart valve disease*':ti,ab,kw OR 'stroke':ti,ab,kw OR 'pulmonary heart disease':ti,ab,kw OR 'diabetes type 1':ti,ab,kw OR 'diabetes type 2':ti,ab,kw OR 'diabetes':ti,ab,kw OR 'tumor*':ti,ab,kw OR 'cancer*':ti,ab,kw OR 'malignant tumor*':ti,ab,kw) AND [embase]/lim

#3. #1 OR #2

#4 ('episodic future thinking':ti,ab,kw OR 'episodic simulation':ti,ab,kw OR 'episodic prospection':ti,ab,kw OR 'mental time travel':ti,ab,kw OR 'prospective thinking':ti,ab,kw OR 'counterfactual thinking':ti,ab,kw OR 'foresight':ti,ab,kw OR 'imagining the future':ti,ab,kw) AND [embase]/lim

#5. #3 AND #4

**Results: 304**

## **Database 4: PsycINFO**

S1

TI ("Chronic Disease" OR "Noncommunicable Diseases" OR "Chronic Illness" OR "Chronic Condition" OR "noninfectious disease*" OR "Cardiovascular Diseases" OR "Hypertension" OR "High Blood Pressure" OR "Coronary Disease" OR "Coronary Artery Disease" OR "Heart Valve Disease*" OR "heart disease*" OR "Stroke" OR "Cerebrovascular Accident" OR "Asthma-Chronic Obstructive Pulmonary Disease Overlap Syndrome" OR "Asthma" OR "COPD" OR "Chronic obstructive pulmonary disease" OR "Chronic Obstructive Lung Disease" OR "Chronic Bronchitis" OR "Pulmonary Arterial Hypertension" OR "Pulmonary Heart Disease" OR "Metabolic Syndrome" OR "Diabetes Mellitus" OR "Diabetes type 1" OR "Diabetes type 2" OR "Obesity" OR "Diabetes" OR "Neoplasms" OR "tumor*" OR "cancer*" OR "malignant tumor*") OR AB ("Chronic Disease" OR "Noncommunicable Diseases" OR "Chronic Illness" OR "Chronic Condition" OR "noninfectious disease*" OR "Cardiovascular Diseases" OR "Hypertension" OR "High Blood Pressure" OR "Coronary Disease" OR "Coronary Artery Disease" OR "Heart Valve Disease*" OR "heart disease*" OR "Stroke" OR "Cerebrovascular Accident" OR "Asthma-Chronic Obstructive Pulmonary Disease Overlap Syndrome" OR "Asthma" OR "COPD" OR "Chronic obstructive pulmonary disease" OR "Chronic Obstructive Lung Disease" OR "Chronic Bronchitis" OR "Pulmonary Arterial Hypertension" OR "Pulmonary Heart Disease" OR "Metabolic Syndrome" OR "Diabetes Mellitus" OR "Diabetes type 1" OR "Diabetes type 2" OR "Obesity" OR "Diabetes" OR "Neoplasms" OR "tumor*" OR "cancer*" OR "malignant tumor*")

S2

TI ("episodic future thinking" OR "episodic simulation" OR "episodic prospection" OR "mental time travel" OR “prospective thinking” OR "counterfactual thinking" OR "foresight" OR "imagining the future") OR AB ("episodic future thinking" OR "episodic simulation" OR "episodic prospection" OR "mental time travel" OR “prospective thinking” OR "counterfactual thinking" OR "foresight" OR "imagining the future")

S3

S1 AND S2

(TI ("Chronic Disease" OR "Noncommunicable Diseases" OR "Chronic Illness" OR "Chronic Condition" OR "noninfectious disease*" OR "Cardiovascular Diseases" OR "Hypertension" OR "High Blood Pressure" OR "Coronary Disease" OR "Coronary Artery Disease" OR "Heart Valve Disease*" OR "heart disease*" OR "Stroke" OR "Cerebrovascular Accident" OR "Asthma-Chronic Obstructive Pulmonary Disease Overlap Syndrome" OR "Asthma" OR "COPD" OR "Chronic obstructive pulmonary disease" OR "Chronic Obstructive Lung Disease" OR "Chronic Bronchitis" OR "Pulmonary Arterial Hypertension" OR "Pulmonary Heart Disease" OR "Metabolic Syndrome" OR "Diabetes Mellitus" OR "Diabetes type 1" OR "Diabetes type 2" OR "Obesity" OR "Diabetes" OR "Neoplasms" OR "tumor*" OR "cancer*" OR "malignant tumor*") OR AB ("Chronic Disease" OR "Noncommunicable Diseases" OR "Chronic Illness" OR "Chronic Condition" OR "noninfectious disease*" OR "Cardiovascular Diseases" OR "Hypertension" OR "High Blood Pressure" OR "Coronary Disease" OR "Coronary Artery Disease" OR "Heart Valve Disease*" OR "heart disease*" OR "Stroke" OR "Cerebrovascular Accident" OR "Asthma-Chronic Obstructive Pulmonary Disease Overlap Syndrome" OR "Asthma" OR "COPD" OR "Chronic obstructive pulmonary disease" OR "Chronic Obstructive Lung Disease" OR "Chronic Bronchitis" OR "Pulmonary Arterial Hypertension" OR "Pulmonary Heart Disease" OR "Metabolic Syndrome" OR "Diabetes Mellitus" OR "Diabetes type 1" OR "Diabetes type 2" OR "Obesity" OR "Diabetes" OR "Neoplasms" OR "tumor*" OR "cancer*" OR "malignant tumor*")) AND (TI ("episodic future thinking" OR "episodic simulation" OR "episodic prospection" OR "mental time travel" OR “prospective thinking” OR "counterfactual thinking" OR "foresight" OR "imagining the future") OR AB ("episodic future thinking" OR "episodic simulation" OR "episodic prospection" OR "mental time travel" OR “prospective thinking” OR "counterfactual thinking" OR "foresight" OR "imagining the future"))

**Results: 58**

## **Database 5: Cochrane Library**

#1	("Chronic Disease" OR "Noncommunicable Diseases" OR "Chronic Illness" OR "Chronic Condition" OR "noninfectious disease*" OR "Cardiovascular Diseases" OR "Hypertension" OR "High Blood Pressure" OR "Coronary Disease" OR "Coronary Artery Disease" OR "Heart Valve Disease*" OR "heart disease*" OR "Stroke" OR "Cerebrovascular Accident" OR "Asthma-Chronic Obstructive Pulmonary Disease Overlap Syndrome" OR "Asthma" OR "COPD" OR "Chronic obstructive pulmonary disease" OR "Chronic Obstructive Lung Disease" OR "Chronic Bronchitis" OR "Pulmonary Arterial Hypertension" OR "Pulmonary Heart Disease" OR "Metabolic Syndrome" OR "Diabetes Mellitus" OR "Diabetes type 1" OR "Diabetes type 2" OR "Obesity" OR "Diabetes" OR "Neoplasms" OR "tumor*" OR "cancer*" OR "malignant tumor*"):ti,ab,kw

#2	("episodic future thinking" OR "episodic simulation" OR "episodic prospection" OR "mental time travel" OR “prospective thinking” OR "counterfactual thinking" OR "foresight" OR "imagining the future") OR AB ("episodic future thinking" OR "episodic simulation" OR "episodic prospection" OR "mental time travel" OR “prospective thinking” OR "counterfactual thinking" OR "foresight" OR "imagining the future"):ti,ab,kw

#3	#1 and #2

**Results: 83**

## **Database 6: CINAHL**

("Chronic Disease" OR "Noncommunicable Diseases" OR "Chronic Illness" OR "Chronic Condition" OR "noninfectious disease*" OR "Cardiovascular Diseases" OR "Hypertension" OR "High Blood Pressure" OR "Coronary Disease" OR "Coronary Artery Disease" OR "Heart Valve Disease*" OR "heart disease*" OR "Stroke" OR "Cerebrovascular Accident" OR "Asthma-Chronic Obstructive Pulmonary Disease Overlap Syndrome" OR "Asthma" OR "COPD" OR "Chronic obstructive pulmonary disease" OR "Chronic Obstructive Lung Disease" OR "Chronic Bronchitis" OR "Pulmonary Arterial Hypertension" OR "Pulmonary Heart Disease" OR "Metabolic Syndrome" OR "Diabetes Mellitus" OR "Diabetes type 1" OR "Diabetes type 2" OR "Obesity" OR "Diabetes" OR "Neoplasms" OR "tumor*" OR "cancer*" OR "malignant tumor*") AND ("episodic future thinking" OR "episodic simulation" OR "episodic prospection" OR "mental time travel" OR “prospective thinking” OR "counterfactual thinking" OR "foresight" OR "imagining the future")

**Results: 69**

## **Database 7: CNKI**

(SU= ('慢性病' + '慢性非传染性疾病' + '慢性消耗性疾病') OR TKA =('心脑血管疾病'+'心血管疾病'+'高血压'+'冠心病'+'高脂血症'+'动脉粥样硬化'+'脑卒中'+'卒中'+'中风'+'心脑血管疾病'+'心脑血管意外'+'慢性心律失常'+'心脏瓣膜疾病'+'心脏病'+'慢性呼吸系统疾病'+'慢性阻塞性肺疾病'+'COPD'+'慢阻肺'+'老慢支'+'慢性阻塞性肺气肿'+'支气管扩张'+'慢性支气管炎'+'哮喘'+'支气管哮喘'+'慢性肺炎'+'肺动脉高压'+'肺源性心脏病'+'肺心病'+'慢性肝炎'+'肝硬化'+'代谢综合征'+'肥胖症'+'糖尿病'+'瘤'+'癌'+'恶性肿瘤')) AND SU=('未来情景思维' + '未来情景想象' + '未来情景思考' + '情景预见')

**Results: 1**

## **Database 8: Wanfang Database**

(主题:(慢性病 or 慢性非传染性疾病 or 慢性消耗性疾病) or 题名或关键词:(心脑血管疾病 or 心血管疾病 or 高血压 or 冠心病 or 高脂血症 or 动脉粥样硬化 or 脑卒中 or 中风 or 心脑血管疾病 or 心脑血管意外 or 慢性心律失常 or 心脏瓣膜疾病 or 心脏病 or 慢性阻塞性肺疾病 or COPD or 慢阻肺 or 老慢支 or 慢性阻塞性肺气肿 or 支气管扩张 or 慢性支气管炎 or 哮喘 or 支气管哮喘 or 慢性肺炎 or 肺动脉高压 or 肺源性心脏病 or 肺心病 or 慢性肝炎 or 肝硬化 or 代谢综合征 or 肥胖症 or 糖尿病 or 瘤 or 癌 or 恶性肿瘤)) and 主题:(未来情景思维 or 未来情景想象 or 未来情景思考 or 情景预见)

**Results: 10**

## **Database 9: CQVIP**

(((((((((((((((((((((((((((((((((((((题名或关键词=慢性病 OR 题名或关键词=慢性非传染性疾病) OR 题名或关键词=慢性消耗性疾病) OR 题名或关键词=心脑血管疾病) OR 题名或关键词=心血管疾病) OR 题名或关键词=高血压) OR 题名或关键词=冠心病) OR 题名或关键词=高脂血症) OR 题名或关键词=动脉粥样硬化) OR 题名或关键词=脑卒中) OR 题名或关键词=中风) OR 题名或关键词=心脑血管疾病) OR 题名或关键词=心脑血管意外) OR 题名或关键词=慢性心律失常) OR 题名或关键词=心脏瓣膜疾病) OR 题名或关键词=心脏病) OR 题名或关键词=慢性阻塞性肺疾病) OR 题名或关键词=COPD) OR 题名或关键词=慢阻肺) OR 题名或关键词=老慢支) OR 题名或关键词=慢性阻塞性肺气肿) OR 题名或关键词=支气管扩张) OR 题名或关键词=慢性支气管炎) OR 题名或关键词=哮喘) OR 题名或关键词=支气管哮喘) OR 题名或关键词=慢性肺炎) OR 题名或关键词=肺动脉高压) OR 题名或关键词=肺源性心脏病) OR 题名或关键词=肺心病) OR 题名或关键词=慢性肝炎) OR 题名或关键词=肝硬化) OR 题名或关键词=代谢综合征) OR 题名或关键词=肥胖症) OR 题名或关键词=糖尿病) OR 题名或关键词=瘤) OR 题名或关键词=癌) OR 题名或关键词=恶性肿瘤) AND (((题名或关键词=未来情景思维 OR 题名或关键词=未来情景想象) OR 题名或关键词=未来情景思考) OR 题名或关键词=情景预见))

**Results: 2**

## **Database 10: SinoMed**

1)	慢性病 or 慢性非传染性疾病 or 慢性消耗性疾病 or 心脑血管疾病 or 心血管疾病 or 高血压 or 冠心病 or 高脂血症 or 动脉粥样硬化 or 脑卒中 or 中风 or 心脑血管疾病 or 心脑血管意外 or 慢性心律失常 or 心脏瓣膜疾病 or 心脏病 or 慢性阻塞性肺疾病 or COPD or 慢阻肺 or 老慢支 or 慢性阻塞性肺气肿 or 支气管扩张 or 慢性支气管炎 or 哮喘 or 支气管哮喘 or 慢性肺炎 or 肺动脉高压 or 肺源性心脏病 or 肺心病 or 慢性肝炎 or 肝硬化 or 代谢综合征 or 肥胖症 or 糖尿病 or 瘤 or 癌 or 恶性肿瘤

2)	未来情景思维 or 未来情景想象 or 未来情景思考 or 情景预见	18

3)	(#2) AND (#1)

**Results: 2**
