# Supplementary material for: Applications of episodic future thinking in patients with chronic diseases: a scoping review
Source: Front Public Health. 2026 Sep 28;14:1914394. doi: 10.3389/fpubh.2026.1914394 (PMC13655094; doi:10.3389/fpubh.2026.1914394)
Supplement: Supplementary file 3 [file Supplementary_file_2.docx]

**Critical Appraisal of the included studies. Mixed Methods Appraisal Tool (MMAT), version 2018**

| **Study** | **Screening questions** | | **2. Quantitative randomised controlled trials** | | | | |  |
| --- | --- | --- | --- | --- | --- | --- | --- | --- |
|  | S1. Are there clear research questions? | S2. Do the collected data allow to address the research questions? | 2.1 Is randomization appropriately performed? | 2.2 Are the groups comparable at baseline? | 2.3 Are there complete outcome data? | 2.4 Are outcome assessors blinded to the intervention provided? | 2.5 Did the participants adhere to the assigned intervention? | MMAT criteria satisfied |
| **Brown et al. 2023** | Yes | Yes | Can’t tell | Yes | No | Can’t tell | Yes | 40% |
| **Epstein et al. 2022(6个月** | Yes | Yes | Yes | Yes | Yes | Yes | No | 80% |
| **Mansouri et al. 2021** | Yes | Yes | Can’t tell | Yes | Yes | No | Yes | 60% |
| **Stein et al. 2021** | Yes | Yes | Yes | Yes | Yes | Yes | Yes | 100% |
| **Chew et al. 2021** | Yes | Yes | Yes | No | Yes | Yes | Can’t tell | 60% |
| **Vaughn et al. 2021** | Yes | Yes | Yes | Yes | Yes | Can’t tell | Yes | 80% |
| **Athamneh et al. 2021** | Yes | Yes | Can’t tell | Yes | Yes | No | Yes | 60% |
| **Craft et al. 2020** | Yes | Yes | Can’t tell | No | Yes | No | Yes | 40% |
| **Segovia et al. 2020** | Yes | Yes | Can’t tell | No | Yes | Yes | Yes | 60% |
| **Bickel et al. 2020** | Yes | Yes | Yes | Yes | Yes | Can’t tell | Can’t tell | 60% |
| **Hollis-Hansen et al. 2020** | Yes | Yes | Can’t tell | Yes | Yes | Can’t tell | No | 40% |
| **Hollis-Hansen et al. 2019** | Yes | Yes | Can’t tell | Yes | Yes | Can’t tell | Yes | 60% |
| **Kakoschke et al. 2018** | Yes | Yes | Yes | Yes | No | Can’t tell | Yes | 60% |
| **Sze et al. 2017** | Yes | Yes | Can’t tell | Yes | Yes | Yes | Yes | 80% |
| **Stein et al. 2017** | Yes | Yes | Can’t tell | Yes | Yes | Yes | Yes | 80% |
| **O'Neill et al. 2016** | Yes | Yes | Can’t tell | Yes | Yes | Can’t tell | Yes | 60% |
| **Daniel et al. 2013（reducing）** | Yes | Yes | Can’t tell | Can’t tell | Yes | Can’t tell | Yes | 40% |

| **Study** | **Screening questions** | | **3. Quantitative nonrandomised** | | | | |  |
| --- | --- | --- | --- | --- | --- | --- | --- | --- |
|  | S1. Are there clear research questions? | S2. Do the collected data allow to address the research questions? | 3.1 Are the participants representative of the target population? | 3.2. Are measurements appropriate regarding both the outcome and intervention (or exposure)? | 3.3. Are there complete outcome data? | 3.4 Are the confounders accounted for in the design and analysis? | 3.5. During the study period, is the intervention administered (or exposure occurred) as intended? | MMAT criteria satisfied |
| **Epstein et al. 2022(imaging** | Yes | Yes | No | Yes | Yes | No | Yes | 60% |
| **Daniel et al. 2013（comparing）** | Yes | Yes | No | Yes | Yes | No | Yes | 60% |

| **Study** | **Screening questions** | | **5. Mixed methods** | | | | |  |
| --- | --- | --- | --- | --- | --- | --- | --- | --- |
|  | S1. Are there clear research questions? | S2. Do the collected data allow to address the research questions? | 5.1 Is there an adequate rationale for using a mixed methods design to address the research question? | 5.2 Are the different components of the study effectively integrated to answer the research question? | 5.3 Are the outputs of the integration of qualitative and quantitative components adequately interpreted? | 5.4 Are divergences and inconsistencies between quantitative and qualitative results adequately addressed? | 5.5 Do the different components of the study adhere to the quality criteria of each tradition of the methods involved? | MMAT criteria satisfied |
| **Chang et al. 2023** | Yes | Yes | Yes | Yes | Yes | No | No | 60% |
